# Relationship Between Xonotlite Crystallite Size and Strength Degradation of Silica-Enriched Oil Well Cement Under 240 °C Curing Conditions

**DOI:** 10.3390/ma19081651

**Published:** 2026-04-20

**Authors:** Guodong Cheng, Lei Chen, Qian Tao, Haoguang Wei, Fuzhu Xie, Jixiang Wang, Jun Lu

**Affiliations:** 1 SINOPEC Research Institute of Petroleum Engineering Co., Ltd., Beijing 102206, China; 2 Key Laboratory of Advanced Civil Engineering Materials of Ministry of Education, School of Materials Science and Engineering, Tongji University, Shanghai 201804, China; 3 School of Chemical and Environmental Engineering, China University of Mining & Technology, Beijing 100083, China; 4 State Pipeline Network Group Energy Storage Technology Co., Ltd., Shanghai 200011, China

**Keywords:** silica-enriched oil well cements, strength degradation, xonotlite, crystallite size, Scherrer equation, high-temperature curing

## Abstract

The strength degradation of silica-enriched oil well cement under high-temperature curing conditions poses a challenge to wellbore integrity. Using the single-peak Scherrer equation, this study evaluated xonotlite crystallite size evolution in cements cured at different setting temperatures. Low-temperature setting (80 °C) maintained stable crystallite size (≈35–36 nm), accompanied by strength gain and pore refinement. High-temperature setting (240 °C) induced crystallite coarsening (up to 40 nm), concurrent with strength degradation and pore coarsening. Similar crystallite sizes led to divergent mechanical performance depending on crystal morphology, highlighting the need for combined size-morphology assessment. These findings identify xonotlite crystallite coarsening as a key indicator of high-temperature cement retrogression.

## 1. Introduction

Portland cement is a fundamental material widely used in oil well cementing operations [1,2]. With the increasing development of deep, ultra-deep, and geothermal wells, downhole temperatures exceeding 110 °C have become the norm, and temperatures above 200 °C are frequently encountered in ultra-deep and geothermal drilling [3]. Under these extreme thermal conditions, calcium silicate hydrate (C-S-H)-the primary cementing phase in set cement-undergoes a progressive phase transformation, leading to coarsening of the cement sheath microstructure and significant strength decline [1,4,5]. This is a critical issue that threatens the long-term sealing integrity of the wellbore. Extensive research indicates that, without countermeasures, neat oil well cement suffers severe compressive strength loss at temperatures above 110 °C due to the transformation of C-S-H into α-dicalcium silicate hydrate (α-C_2_SH), which possesses inferior mechanical properties [4]. The addition of silica sand to cement to suppress this strength decline has become a widely adopted standard practice in the industry [1,6,7,8,9]. The fundamental mechanism involves reducing the calcium-to-silica molar ratio in the system to promote the formation of low-Ca/Si crystalline phases such as tobermorite and xonotlite [1]. However, when set under ultra-high-temperature conditions (≥200 °C), the effectiveness of silica addition is limited [1]. A recent study indicates that even the optimized silica-enriched systems, when set and cured under ultra-high-temperature conditions (≥200 °C), still exhibit significant long-term strength decline due to microstructural coarsening, which is caused by the progressive crystallization and coarsening of hydration products during prolonged curing [1,2]. This phenomenon poses a severe challenge to the durability of cement sheaths in ultra-deep wells and high-temperature geothermal applications.

Indeed, setting temperature has a significant impact on the strength and stability of silica-enriched oil well cement systems [10]. Cement slurry set at a low temperature, such as 80 °C for 24 h (initial hardening), and then cured at a high temperature, such as 240 °C, for up to 28 days (simulating heavy oil thermal recovery cementing conditions), exhibited sustained strength gain during the long-term curing period [4,5,8,11,12], whereas the slurry that was both set and cured at high temperature (simulating ultra-deep well cementing conditions) exhibited pronounced strength decline. Quantitative XRD analysis further revealed that C-S-H gel, as the primary contributor to strength, plays a crucial role in high-temperature strength stability through its early transformation into xonotlite [1]. Specifically, the low-temperature setting system produced a large amount of xonotlite at 3 days and maintained a stable content thereafter, whereas in the high-temperature setting system, C-S-H gel continued to transform into xonotlite throughout the entire long-term curing process [10].

Xonotlite is the key product of this transformation and serves as the typical hydration phase in silica-enriched oil well cement under ultra-high-temperature curing conditions, formed by the crystallization of C-S-H gel [13]. Studies have shown that in the low-temperature setting system, xonotlite exhibits a dense, ordered fibrous structure; in contrast, in the high-temperature setting system, xonotlite typically appears as coarse, needle-like crystals with relatively loose packing, which adversely affects the strength and stability of the set cement [10]. More importantly, prolonged curing under ultra-high-temperature conditions leads to coarsening of xonotlite crystallites, characterized by an increase in average crystallite diameter and a decrease in specific surface area [14]. This coarsening phenomenon is considered one of the primary causes of the long-term mechanical performance degradation of silica-enriched oil well cement at high temperatures [15,16].

X-ray diffraction (XRD) peak shape analysis provides a well-established method for quantifying crystallite size [17,18,19,20,21,22,23,24]. The Scherrer equation, first proposed in 1918s, relates the width of a diffraction peak to the crystallite size perpendicular to the diffracting plane: D = Kλ/(βcosθ), where D is the crystallite size, K is the Scherrer constant, λ is the X-ray wavelength, β is the full width at half maximum (FWHM) of the diffraction peak after instrumental broadening correction, and θ is the Bragg diffraction angle [17,25,26,27,28,29]. Due to its simplicity and applicability to individual diffraction peaks, the Scherrer equation has been widely used for determining crystallite size in cement-based materials [30,31,32]. However, the Scherrer equation assumes that peak broadening stems solely from finite crystallite size, neglecting the contribution of lattice microstrain, which may lead to an overestimation of crystallite size for strain-broadened peaks [33,34]. The Williamson-Hall (W-H) method attempts to address this limitation based on the assumption of material isotropy [34,35,36]; however, this assumption may not hold for some mineral phases in cement-based materials due to their inherent microstructural heterogeneity and anisotropy [37]. By plotting the relationship between βcosθ and 4sinθ for multiple diffraction peaks, the W-H method allows the average crystallite size to be determined from the y-intercept of the linear fit, and the lattice microstrain from the slope: βcosθ = Kλ/D + 4εsinθ [34].

Currently, the Scherrer equation has been applied to quantitatively analyze the crystallite size of tobermorite in silica-enriched oil well cement cured at 200 °C, with a reported stable value of approximately 8–9 nm [1]. However, the crystallite size of xonotlite, which becomes the dominant crystalline phase above approximately 200 °C, has not yet been quantitatively characterized. In systems cured at 240 °C, xonotlite constitutes the primary phase, while tobermorite exists only as a minor transient intermediate [10]; the crystallite growth behavior of xonotlite is expected to have a significant impact on long-term strength evolution. In this study, the crystallite size of xonotlite was quantified using both the W-H method and the Scherrer equation, and the effectiveness of these methods was compared. The effects of two different setting temperatures on the crystallite growth of xonotlite were investigated, and a correlation analysis was performed between the crystallite growth trends and strength decline.

## 2. Materials and Experimental Methods

### 2.1. Materials

All experiments were based on API Class G oil-well cement with standardized composition, which served as the internationally recognized benchmark oil-well cement; the type of silica sand used was selected based on preliminary optimization experiments targeting high-temperature performance. Their chemical compositions and particle size distributions are presented in Table 1 and Table 2, respectively. The chemical compositions of cement and silica sand were determined using X-ray fluorescence (XRF) spectrometry (Malvern PANalytical B.V., AXIOS, Almelo, The Netherlands) in accordance with the Chinese national standard GB/T 176-2017 [38]. Particle size distribution was measured using a laser particle size analyzer (Mastersizer 2000, Malvern Instruments, Malvern, UK). Based on the particle size distribution data, the surface area was calculated and reported by the instrument software, assuming spherical particle geometry. The free lime content was determined in accordance with the Chinese national standard GB/T 176-2017 [38]. The water used in the experiment was ordinary tap water.

### 2.2. Formulation Design and Slurry Preparation

Table 3 presents the formulation design used in this study. All slurries were designed with a final density of 1.9 g/cm^3^. In the T1 and T2 systems, the silica sand content was 50% by weight of cement (BWOC), which was a dosage commonly used in ultra-high-temperature cementing operations and had also been adopted in several existing studies [39,40]. The essential distinction was that T1 contained no high-temperature retarder to ensure rapid setting at low temperature (80 °C), while T2 included a sufficient amount of retarder to ensure a thickening time of longer than 4 h—that is, longer than the time required for the curing autoclave to heat up to 240 °C. This guaranteed that the cement slurry remained in a fluid state before reaching the specified curing temperature, thereby enabling it to set under high-temperature conditions. The silica sand content in the T3 system was increased to 110% BWOC specifically to investigate the effect of high silica sand content on the stability of set cement, constituting a critical comparative experimental point. Slurry T1 was a low-temperature setting system, while slurries T2 to T3 were high-temperature setting systems, with thickening times all longer than the heating-up time of the curing autoclave. Except for the suspending agent and dispersant, which were solid powders, all other admixtures had a solid content of 80%, supplied by Sinopec Petroleum Engineering Technology Research Institute Co., Ltd., Beijing, China. In slurry T1, the dosages of the suspending agent and defoamer were 1% and 1% (BWOC), respectively, and no other admixtures were added. In slurries T2 and T3, the dosages of the dispersant (USZ), retarder (SCR-4 and SCR-7), water-loss reducer (240 W), suspending agent and defoamer were 1%, 8%, 1.8%, 4%, 1% and 1% (BWOC), respectively. All cement pastes were prepared strictly in accordance with API Recommended Practice 10B-2 and subsequently poured into square stainless steel molds measuring 5 cm × 5 cm × 5 cm.

T1 was first set at 80 °C for 24 h, then demoulded and cured at 240 °C/20 MPa until the designated test age, representing the low-temperature setting system. T2 and T3 were directly set and cured at 240 °C/20 MPa until the designated test age, representing the high-temperature setting systems. It should be emphasized that the “Set” refers to the initial hardening process at the specified temperature, and the “Curing” refers to the subsequent long-term isothermal holding period at 240 °C/20 MPa until the designated test age (3 d or 28 d).

After demoulding, all specimens were transferred to identical high-temperature curing conditions (240 °C/20 MPa) and cured continuously until the specified test age. The pressure “20 MPa” was the pressure set for the high-temperature, high-pressure curing autoclave, which approximates downhole high pressure. Furthermore, all of the above mix proportions were based on cement mass, which was standard practice in cement slurry design for the cementing industry. To clarify this design, this study also included expressions commonly used in the concrete industry. Based on the cement content of each formulation in Table 4, all admixture dosages originally given in BWOC were converted to their specific mass per cubic meter of slurry. In slurry T1, this included a suspending agent at 9.09 kg/m^3^ and a defoamer at 9.09 kg/m^3^, with no other admixtures added. In slurry T2, the dosages of the dispersant (USZ), retarder (SCR-4 and SCR-7), water-loss reducer (240 W), suspending agent and defoamer were 8.89, 71.13, 16.00, 35.56, 8.89, 8.89 (kg/m^3^), respectively, and in slurry T3, the dosages of the dispersant (USZ), retarder (SCR-4 and SCR-7), water-loss reducer (240 W), suspending agent and defoamer were 6.49, 51.91, 11.68, 25.95, 6.49, 6.49 (kg/m^3^), respectively. Notably, previous studies have indicated that under the specified ultra-high-temperature curing condition (≥200 °C), the hydration reaction of cement in set cement was largely completed. Consequently, the effect of these chemical admixtures (e.g., retarders) on the thermal stability of set cement during long-term service (≥3 days) under ultra-high-temperature environments was considered negligible [1,10,13].

### 2.3. Test Method

The compressive strength test was conducted in accordance with GB/T 19139-2012 standard [41], using an unconfined loading method. The specimens were 5 cm cubes, and the loading rate was 1.195 kN/s. A TG-300B load frame (Shenyang Tiger Petroleum Instruments Manufacturing Co., Ltd., Shenyang, China.) was employed for mechanical loading at a constant rate of 2.4 kN/s until specimen failure.

XRD data were collected using a Panalytical diffractometer (Malvern PANalytical B.V., Almelo, The Netherlands; Model Aeris with a 600 W Cu-anode source, λ = 1.541 Å;) operated at 40 kV and 15 mA. All scans were measured over an angular range of 7° to 70° (2θ angle) with a 0.01° 2θ step size and scanning time per step of 75.22 s, resulting in a total measurement time of about 30 min per scan.

In the mercury intrusion porosimetry (MIP) test, cylindrical samples were first cut into thin slices and further crushed into small pieces with a maximum size of less than 5 mm, yielding a total sample mass of approximately 1 g. Testing was performed using a Quantachrome mercury intrusion porosimeter (Model PM 33, Quantachrome Instruments, Boynton Beach, FL, USA), with a maximum pressure of 32,000 psi (~ 220 MPa) applied during the experiment.

**Figure 1:**
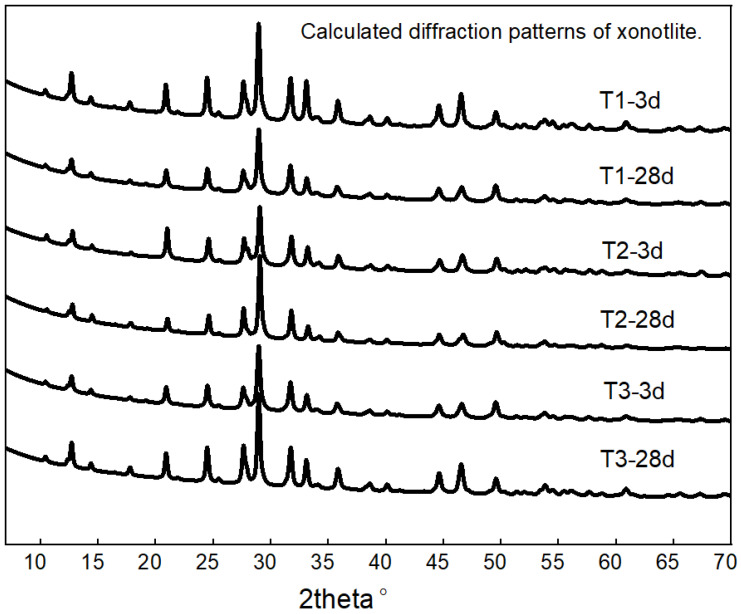
Calculated diffraction patterns of xonotlite isolated from the multiphase cement matrix.

The crystallite size of quantitative analysis was performed based on the Rietveld refinement method, as described in our previous study [10]. To isolate the diffraction contribution of xonotlite from the multiphase cement matrix, its calculated diffraction pattern was generated based on the refined crystal structure model and scale factor of xonotlite. This approach effectively deconvolves overlapping diffraction peaks, yielding a set of diffraction patterns corresponding exclusively to xonotlite (Figure 1), thereby establishing a foundation for subsequent microstructural analysis. It should be noted that inherent discrepancies exist between the calculated and measured diffraction patterns, which are intrinsic to the Rietveld method. Nevertheless, the overall goodness-of-fit was satisfactory [10], indicating that the calculated pattern accurately reflects the actual diffraction characteristics of xonotlite.

The crystallite size of xonotlite was quantitatively analyzed using the single-peak Scherrer equation. Following common practice in XRD crystallite size analysis, the strongest (0$\bar{2}$1) diffraction peak (2θ ≈ 28.92°, Cu Kα radiation) was selected as the primary peak for all samples. To further verify the reliability of the observed crystallite size trend, two additional diffraction peaks of xonotlite, namely the (100) peak (2θ ≈ 12.60°) and the ($\bar{1}$02) peak (2θ ≈ 24.43°), were also analyzed using the same single-peak Scherrer method. Peak shape analysis of the extracted xonotlite diffraction peaks was performed using HighScore Plus 5.0 software (Malvern Panalytical, Almelo, The Netherlands) equipped with the COD (Crystallography Open Database). The reference pattern for xonotlite (COD ID: 96-900-8439) was used for phase identification. Peak fitting was carried out using the pseudo-Voigt function to obtain accurate peak positions and full width at half maximum (FWHM) values. Instrumental broadening correction was performed using a standard reference material (lanthanum hexaboride, LaB_6_, NIST SRM 660c, National Institute of Standards and Technology, Gaithersburg, MD, USA). The Scherrer equation is expressed as follows:

D = Kλ/(βcosθ)

where D is the crystallite size, K is the shape factor (taken as 0.9), λ is the X-ray wavelength (1.5406 Å), β is the FWHM corrected for instrumental broadening, and θ is the Bragg diffraction angle.

To evaluate the potential contribution of microstrain to peak broadening, the Williamson-Hall (W-H) method was also employed for comparative analysis. According to the classical W-H equation, a W-H plot was constructed by plotting βcosθ against 4sinθ, and linear regression was performed:

βcosθ = Kλ/D + 4ε sinθ

The strain-corrected crystallite size was obtained from the intercept, and the microstrain ε was estimated from the slope.

## 3. Test Results and Discussion

### 3.1. Evolution of Xonotlite Crystallite Size

**Figure 2:**
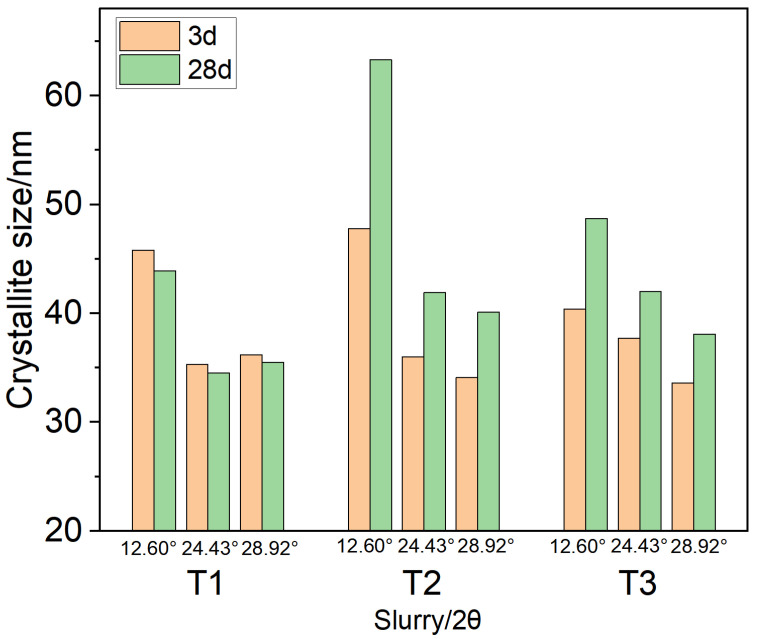
Crystallite size of xonotlite evolution with curing age for different formulations, determined from three diffraction peaks (12.60°, 24.43°, and 28.92°) using the Scherrer equation.

**Figure A1:**
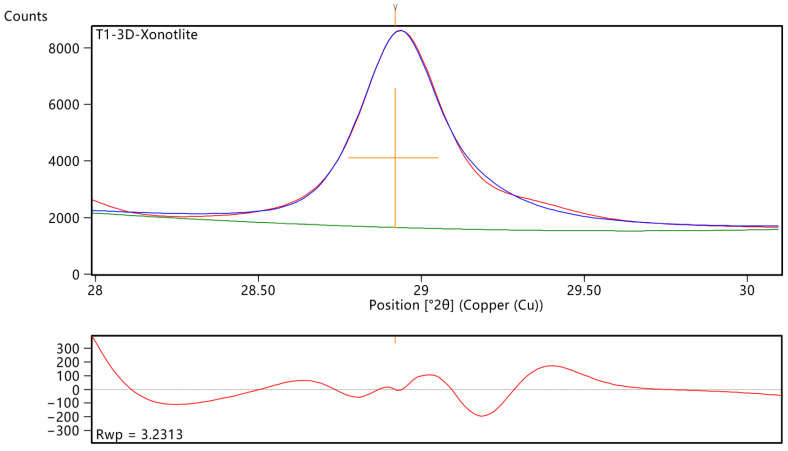
Single-peak fitting patterns of the 28.9° diffraction peak for slurry T1 (3 d).

**Figure A2:**
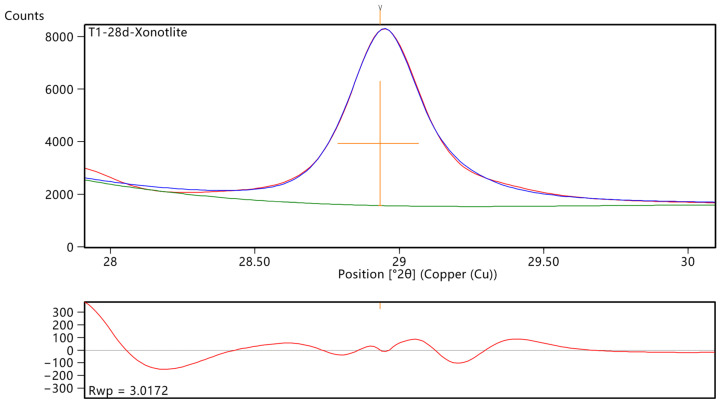
Single-peak fitting patterns of the 28.9° diffraction peak for slurry T1 (28 d).

**Figure A3:**
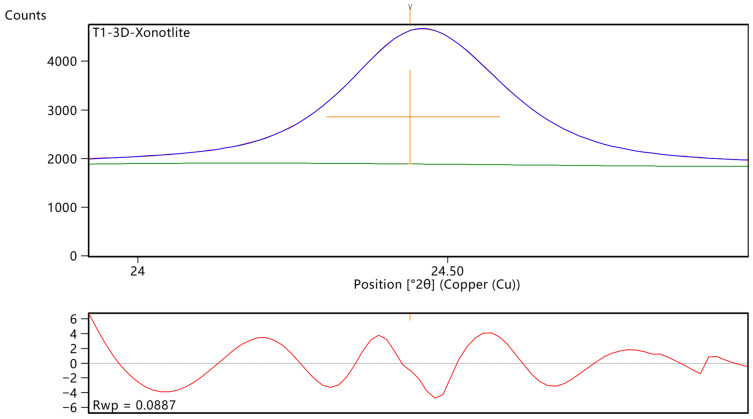
Single-peak fitting patterns of the 24.5° diffraction peak for slurry T1 (3d).

**Figure A4:**
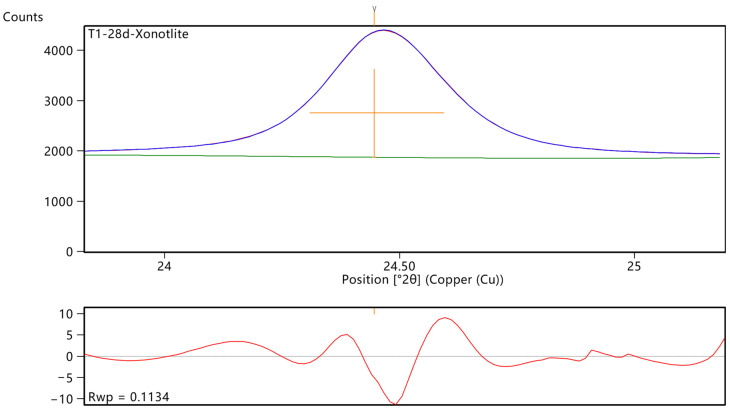
Single-peak fitting patterns of the 24.5° diffraction peak for slurry T1 (28 d).

**Figure A5:**
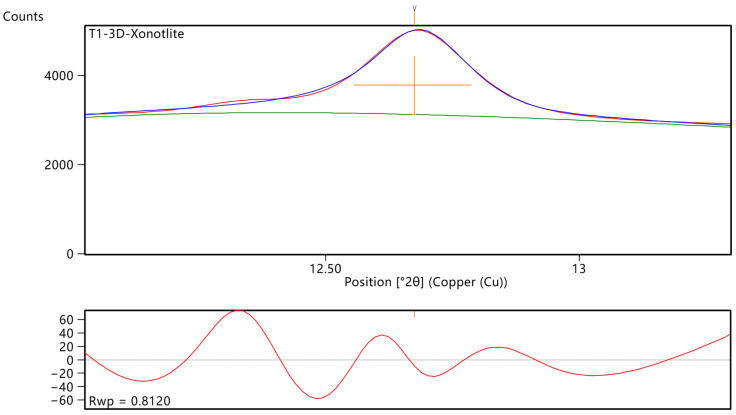
Single-peak fitting patterns of the 12.6° diffraction peak for slurry T1 (3 d).

**Figure A6:**
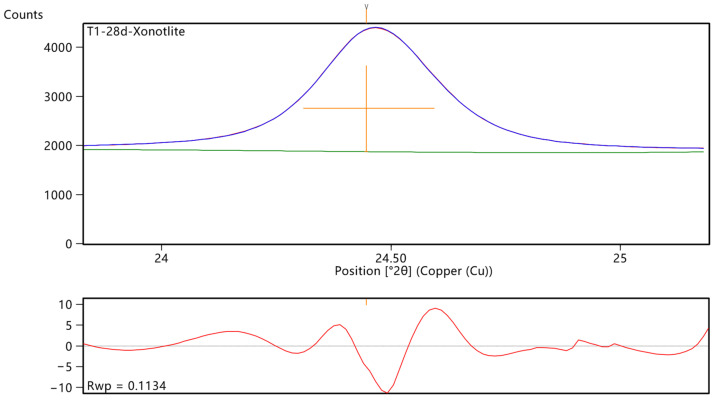
Single-peak fitting patterns of the 12.6° diffraction peak for slurry T1 (28 d).

**Figure A7:**
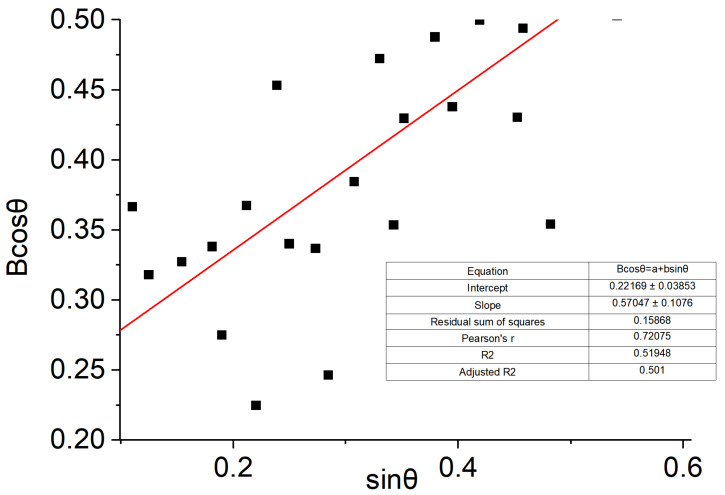
Williamson–Hall plot of xonotlite for slurry T1 (3 d).

**Figure A8:**
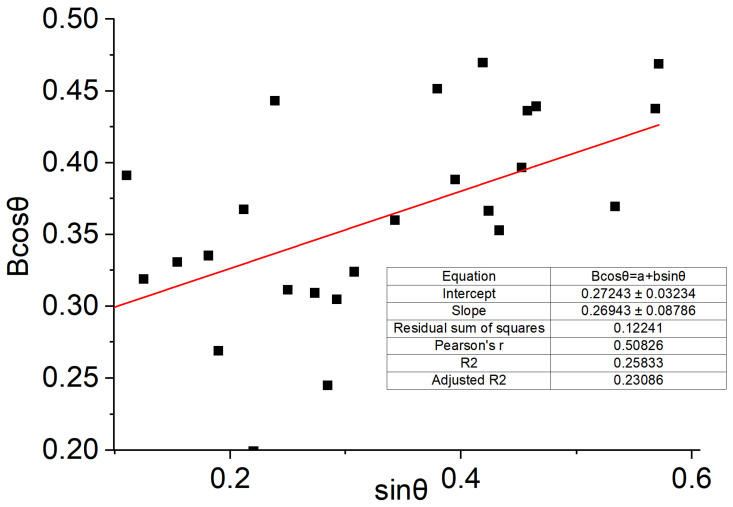
Williamson–Hall plot of xonotlite for slurry T1 (28 d).

The application of the isotropic Williamson-Hall method resulted in severely inadequate fitting quality after validation. Representative fitting results were shown in Figure A7 and Figure A8 in Appendix A, with the highest coefficient of determination (R^2^) being only approximately 0.52, even after removing the outliers. Therefore, this method was inapplicable. In this study, the single-peak Scherrer equation, which avoids the influence of anisotropy, was adopted for calculation, as presented in Table 5 and Figure 2. The quality of the single-peak fitting for the Scherrer equation was evaluated using the weighted residual factor (RWP), where smaller RWP values indicated better fitting performance. In this study, the RWP values of the fittings ranged from 0.0887 to 3.2313, demonstrating exceptionally high precision in peak profile fitting. The extracted peak positions and full width at half maximum (FWHM) data were therefore reliable. Consequently, the crystallite sizes calculated based on these parameters are of high confidence. Detailed analytical data and representative single-peak fitting results were provided in Table A1, Table A2 and Table A3 and Figure A1, Figure A2, Figure A3, Figure A4, Figure A5 and Figure A6 in Appendix A. Specifically, the crystallite sizes derived from the three crystallographic planes of xonotlite in different systems exhibited consistent trends across varying curing times, confirming the reliability of crystallite size evolution. Meanwhile, within the same system, the differences in crystallite growth rates among the different crystallographic planes of xonotlite were observed, reflecting the anisotropic nature of xonotlite and explaining the poor fitting quality of the Williamson-Hall method in this system.

Following common practice in XRD crystallite size analysis, the strongest (222) diffraction peak of xonotlite was selected as the primary reflection. The calculation results showed that for system T1, the crystallite sizes at 3 d and 28 d were 36.2 nm and 35.5 nm, respectively, remaining relatively stable, indicating that the low-temperature setting allows xonotlite to form a stable initial crystal structure, even during subsequent long-term high-temperature curing at 240 °C. In contrast, the high-temperature curing systems T2 and T3 both exhibited comparable crystallite growth behavior of xonotlite. Specifically, T2 increased from 34.1 nm to 40.1 nm, representing a growth of 17.6%, while T3 increased from 33.6 nm to 38.1 nm, representing a growth of 13.4%. The crystallite sizes and growth magnitudes were relatively close, indicating that the increase in the dosage of silica sand content did not alter the crystallite growth of xonotlite. These results collectively suggest that low-temperature curing inhibits the coarsening of xonotlite crystallites, whereas high-temperature curing promotes crystallite growth, which is detrimental to strength stability.

### 3.2. Comparison of Crystallite Size Evolution and Compressive Strength

Based on the evolution data of xonotlite (2θ ≈ 28.92°) crystallite size and compressive strength presented in Table 6, the following observations can be made. For the low-temperature setting system T1, the xonotlite crystallite size remained stable during curing from 3 d to 28 d, accompanied by a significant increase in compressive strength from 18.27 MPa to 31.13 MPa. Meanwhile, SEM observations [10] revealed that the system generated substantial amounts of fibrous and orderly arranged xonotlite at 3 days, and this structure remained stable throughout the 28-day curing period, accompanied by a relatively dense microstructure. Together, these two microstructural features provided a structural basis for the continuous strength gain. In contrast, the high-temperature setting systems T2 and T3 exhibited a consistent trend between crystallite coarsening and strength degradation. For T2, the crystallite size was 34.1 nm at 3 days, with a compressive strength of 22.70 MPa, higher than that of T1 at the same age. However, after 28 days of curing, the crystallite size coarsened to 40.1 nm, while the compressive strength decreased to 16.16 MPa. Notably, despite a difference of only 4.6 nm in crystallite size between T1 and T2 at 28 d, the difference in compressive strength reached nearly 15 MPa. Meanwhile, SEM observations [10] showed that xonotlite in T2 exhibited a coarse acicular morphology with loose stacking and poor interlocking between crystals. This morphological feature is consistent with the trend of crystallite coarsening, collectively reflecting the deterioration of the microstructure.

Similarly, for T3, the crystallite size increased markedly from 33.6 nm to 38.1 nm, accompanied by a strength decrease from 28.05 MPa to 22.12 MPa. Taken together, these observations suggest that, in contrast to low-temperature settings, the continuous growth of xonotlite crystallite size under high-temperature setting conditions may be a contributing factor to the strength degradation of high-temperature cured set cement.

### 3.3. MIP Analysis and Comparison of Crystallite Size Evolution and Pore Structure

**Figure 3:**
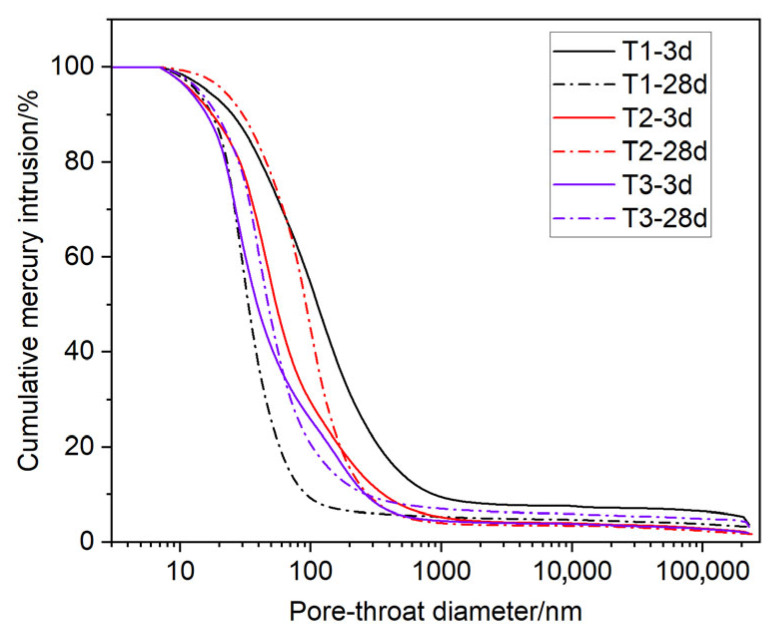

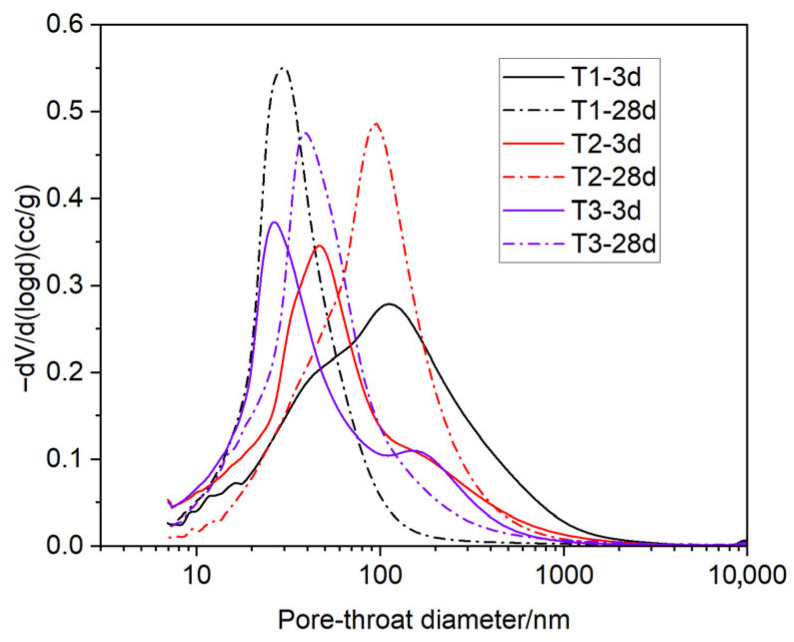
MIP test results of slurries T1 to T3 cured from 3 d to 28 d.

The MIP results for silica-enriched oil well cement after 3 and 28 days of curing at 240 °C are presented in Figure 3. In the low-temperature setting system T1, the cumulative mercury intrusion curve shifted to the left between 3 and 28 days, indicating substantial pore refinement. By contrast, the curves for the high-temperature setting systems T2 and T3 shifted to the right. Accordingly, the pore-size distribution of system T1 narrowed considerably from 7–1700 nm to 7–400 nm, and its median pore size decreased from 104 nm to 29 nm, cured from 3 d to 28 d. Although the pore-size distributions of T2 and T3 remained relatively stable (7–960 nm) over 28 days, their median pore sizes increased from 47 nm to 96 nm for T2 and from 25 nm to 40 nm for T3. This suggested that even with a higher silica-sand content, pore-structure coarsening was not reversed but only attenuated. Collectively, the pore-throat evolution data showed that after extended curing at 240 °C, the high-temperature setting systems underwent clear microstructural coarsening, whereas the low-temperature setting system achieved progressive densification of the pore-throat network. This evolution in pore structure aligns well with the compressive strength development trends shown in Table 6. The continuous densification of the low-temperature setting system T1 provided structural support for its steady increase in strength, whereas the pore coarsening observed in the high-temperature setting systems T2 and T3 directly corresponded to their macroscopic strength degradation.

Table 7 shows the comparison of pore structure for different slurries cured from 3 d to 28 d. According to Shen et al. [42], pores larger than 50 nm are generally considered harmful because they significantly impair the mechanical strength and impermeability of cement-based materials. In contrast, pores smaller than 50 nm have a much less detrimental effect. For the low-temperature setting system T1, during the curing period from 3 d to 28 d, the total porosity decreased from 50.39% to 43.83%, and the proportion of pores larger than 50 nm significantly decreased from 37.52% to 10.67%, exhibiting a refined and densified pore structure, consistent with the stable crystallite size. In contrast, for the high-temperature setting system T2, the total porosity increased from 39.16% to 47.81%, and the proportion of pores larger than 50 nm increased from 20.98% to 36.53%, characterized by pore structure coarsening, in line with the crystallite coarsening trend. The high-temperature setting system T3 similarly exhibited pore structure coarsening: the total porosity increased from 40.71% to 45.98%, and the proportion of pores larger than 50 nm increased from 16.50% to 22.72%. These comparative results suggest that low-temperature setting inhibits the coarsening of xonotlite crystallites and the deterioration of pore structure, which is associated with strength enhancement. In contrast, under high-temperature setting conditions, the continuous growth of xonotlite crystallites occurs concomitantly with pore structure coarsening, which is associated with strength degradation of set cement. Furthermore, the comparison between T2 and T3 systems suggests that although increasing the silica sand content can alleviate the extent of pore coarsening to some degree, it does not fundamentally reverse the microstructural deterioration trend under high-temperature curing conditions.

## 4. Discussion

Low-temperature setting (80 °C) effectively suppressed the coarsening of xonotlite crystallites, facilitating pore structure refinement and sustained strength gain. In contrast, high-temperature setting (240 °C) promoted continuous crystallite growth, accompanied by pore structure coarsening and strength degradation. This differential behavior can be attributed to the kinetics of Ostwald ripening [43], which was accelerated at elevated temperatures. In high-temperature systems, grain boundary migration can engulf or merge pores, explaining the concurrent coarsening of crystallites and pore structure observed in T2 and T3. The presence of excess silica in T3 appears to moderately suppress this process, suggesting that local supersaturation of the liquid phase may influence the growth kinetics. However, the effect was palliative rather than preventive, as microstructural degradation remains evident. Several limitations should be acknowledged. The limited sample size precludes statistical validation of the observed trends; thus, the identified relationships are qualitative. Additionally, the analysis relies on two discrete time points, which may not fully capture the temporal dynamics of microstructural evolution. Future work incorporating higher temporal resolution and quantitative three-dimensional characterization would be valuable to further elucidate the underlying mechanisms. Despite these limitations, the consistent trends across XRD, MIP, SEM, and mechanical testing support the conclusion that controlling the crystallite size growth of xonotlite is a key factor in mitigating the strength degradation of high-temperature cured cement stone.

## 5. Conclusions

Based on a systematic investigation of the evolution of xonotlite crystallite size, pore structure, and compressive strength in silica-enriched oil well cement cured at 240 °C, the following conclusions are drawn:

1. The single-peak Scherrer equation, applied to the strongest (222) diffraction peak, provided a reliable method for evaluating crystallite size evolution in anisotropic xonotlite systems, circumventing the limitations of the Williamson-Hall method, which was established on the assumption of isotropy. Due to the anisotropic nature of xonotlite, the latter exhibits poor fitting quality (R^2^ < 0.52).
2. Setting temperature dictates crystallite growth. Low-temperature setting (80 °C) suppressed coarsening, while high-temperature setting promoted it (Table 5), highlighting the critical role of early thermal history on microstructural stability.
3. Crystallite size, pore structure, and strength evolved together. Stable crystallite size with pore refinement and strength gain, while crystallite coarsening with pore coarsening and strength loss, indicated that microstructural coarsening (crystallite growth plus pore enlargement) drove strength degradation in high-temperature cured cement.
4. Although increasing the silica sand content (T3 vs. T2) moderately alleviated the extent of crystallite coarsening and pore structure degradation, it did not fundamentally reverse the microstructural deterioration trend under prolonged high-temperature curing conditions.
5. Controlling the crystallite growth of xonotlite can serve as an important means to mitigate the strength degradation of high-temperature silica-enriched oil well cement systems.

## Figures and Tables

**Table 1:** Main oxides of bulk materials used (%).

Oxide Name	Class G Cement	Silica
Al_2_O_3_	3.00	1.028
CaO	63.10	1.379
Fe_2_O_3_	5.13	0.786
TiO_2_	-	-
K_2_O	0.45	0.257
MgO	2.84	0.412
Na_2_O	0.33	0.176
SO_3_	3.76	0.310
P_2_O_5_	0.043	0.014
SiO_2_	20.01	95.640
Free Lime	1.360	-

**Table 2:** Summary of material properties: particle size and specific gravity.

Material	D10 μm	D50 μm	D90 μm	Surface Area m^2^/kg	Specific Gravity
silica	6.71	46.08	108.33	278	2.67
Class G cement	1.93	13.12	42.92	522	3.25

**Table 3:** Formulation design of dry blend compositions (proportions by weight of cement, BWOC).

Formulation	Cement	Silica	Added Water
T1	100	50	57.0
T2	100	50	46.9
T3	100	110	66.0

**Table 4:** Mix proportions of cement slurries.

Formulation	Cement (kg/m^3^)	Silica (kg/m^3^)	Water-to-Solid Ratio (by Mass)
T1	909.00	454.55	0.58
T2	889.10	444.55	0.40
T3	648.91	713.79	0.38

Note: All admixtures are aqueous solutions with 80% water content. The total water content includes water from admixtures. The water-to-solid ratio is calculated as total water/(cement + silica).

**Table 5:** Crystallite size results of xonotlite calculated by the Scherrer equation.

Slurry	Crystal Plane (hkl)	Peak Pos. [°2θ]	Crystallite Size/nm	
			3 d	28 d
	(100)	12.60	45.8	43.9
T1	($\bar{1}$02)	24.43	35.3	34.5
	(0$\bar{2}$1)	28.92	36.2	35.5
	(100)	12.60	47.8	63.3
T2	($\bar{1}$02)	24.43	36	41.9
	(0$\bar{2}$1)	28.92	34.1	40.1
	(100)	12.60	40.4	48.7
T3	($\bar{1}$02)	24.43	37.7	42.0
	(0$\bar{2}$1)	28.92	33.6	38.1

**Table 6:** Comparison of xonotlite crystallite size (2θ ≈ 28.92°) and compressive strength for different slurries cured for 3 d to 28 d.

Slurry	Curing Age (Day)	Crystallite Size (nm)	Compressive Strength (MPa)
T1	3	36.2	18.3
T1	28	35.5	31.1
T2	3	34.1	22.7
T2	28	40.1	16.2
T3	3	33.6	28.1
T3	28	38.1	22.1

**Table 7:** Comparison of xonotlite crystallite size (2θ ≈ 28.92°) and pore structure for different slurries cured for 3 d to 28 d.

Slurry	Curing Age (Day)	Crystallite Size (nm)	Total Porosity/%	Proportion of Pore Sizes > 50 nm
T1	3	36.2	50.4	37.5
T1	28	35.5	43.8	10.7
T2	3	34.1	39.2	21.0
T2	28	40.1	47.8	36.5
T3	3	33.6	40.7	16.5
T3	28	38.1	46.0	22.7

## Data Availability

The original contributions presented in this study are included in the article. Further inquiries can be directed to the corresponding author.

## Appendix A

**Table A1:** Crystallite Size Results Calculated by the Scherrer Equation (28.92°).

Slurry/Curing Days	Bobs. [°2θ]	Bstd. [°2θ]	Peak Pos. [°2θ]	B Struct. [°2θ]	Crystallite Size	
					[Å]	[nm]
T1-3d	0.277	0.050	28.920	0.226	362	36.2
T1-28d	0.281	0.050	28.920	0.231	355	35.5
T2-3d	0.291	0.050	28.920	0.241	341	34.1
T2-28d	0.255	0.050	28.920	0.205	401	40.1
T3-3d	0.294	0.050	28.920	0.244	336	33.6
T3-28d	0.265	0.050	28.920	0.215	381	38.1

**Table A2:** Crystallite Size Results Calculated by the Scherrer Equation (24.43°).

Slurry/Curing Days	Bobs. [°2θ]	Bstd. [°2θ]	Peak Pos. [°2θ]	B Struct. [°2θ]	Crystallite Size	
					[Å]	[nm]
T1-3d	0.281	0.050	24.430	0.230	353	35.3
T1-28d	0.286	0.050	24.430	0.236	345	34.5
T2-3d	0.276	0.050	24.430	0.226	360	36.0
T2-28d	0.244	0.050	24.430	0.194	419	41.9
T3-3d	0.266	0.050	24.300	0.215	377	37.7
T3-28d	0.243	0.050	24.300	0.193	420	42.0

**Table A3:** Crystallite Size Results Calculated by the Scherrer Equation (12.60°).

Slurry/Curing Days	Bobs. [°2θ]	Bstd. [°2θ]	Peak Pos. [°2θ]	B Struct. [°2θ]	Crystallite Size	
					[Å]	[nm]
T1-3d	0.225	0.050	12.600	0.175	458	45.8
T1-28d	0.232	0.050	12.600	0.182	439	43.9
T2-3d	0.217	0.050	12.600	0.167	478	47.8
T2-28d	0.176	0.050	12.600	0.126	633	63.3
T3-3d	0.248	0.050	12.600	0.198	404	40.4
T3-28d	0.214	0.050	12.600	0.164	487	48.7
